# CHANGES IN DEPRESSIVE SYMPTOMS ACROSS MIDDLE AND OLDER ADULTHOOD IN THE ORANJ BOWL LONGITUDINAL STUDY OF AGING

**DOI:** 10.1093/geroni/igae098.2667

**Published:** 2024-12-31

**Authors:** Kaite Yang, Christine Ferri, Joan Girgus

**Affiliations:** Stockton University, Galloway, New Jersey, United States; Stockton University, Galloway, New Jersey, United States; Princeton University, Princeton, New Jersey, United States

## Abstract

Life course fluctuations in depressive symptoms are important to document and understand. Previous research has suggested a U-shaped pattern in depressive symptoms across the lifespan, with more symptoms in younger and older adulthood compared to middle adulthood (Sutin et al., 2013). The present study used hierarchical linear modeling to examine changes in depressive symptoms in middle and older adulthood and the degree to which participants’ age at baseline affected symptom trajectories across 13 years. Participants were a representative sample of community-dwelling adults in New Jersey aged 50-74 (M=60.53) at baseline (ORANJ BOWL; Pruchno et al., 2010). N=4266 participants (64.3% female) who completed at least two waves of data collection from five timepoints were included in this study (T1: 2006-2008; T3: 2011; T4: 2013-2015; T5: 2015-2017; T6: 2017-2019). The trajectory of depressive symptoms (CESD-R-10) was best captured by a cubic model wherein symptoms peaked at T3, decreased, and then increased again after T4. There were significant intercept, linear, quadratic, and cubic terms for the effect of age as a continuous variable on depressive symptoms. Participants who were younger at the start of the study had more symptoms initially and then flatter trajectories compared to participants who were older. Participants who were older started with less symptoms but had a steeper increase in symptoms over time. These findings suggest that age-dependent fluctuations as well as impacts of and recovery from exogenous events like the Great Recession are reflected in depressive symptom trajectories in older adults (Pruchno et al., 2021).

